# SARS-CoV-2 infection in high-risk children following tixagevimab–cilgavimab (Evusheld) pre-exposure prophylaxis: a single-center observational study

**DOI:** 10.3389/fonc.2023.1229655

**Published:** 2023-08-03

**Authors:** Diego R. Hijano, Jose A. Ferrolino, Elizabeth G. Swift, Carolyn A. Michaels, Anita Max, Randall T. Hayden, Joshua Wolf, Ronald H. Dallas, William L. Greene, Julie L. Richardson, Hana Hakim, Ted H. Morton, Shane J. Cross

**Affiliations:** ^1^ Department of Infectious Diseases, St. Jude Children Research Hospital, Memphis, TN, United States; ^2^ Center for Advanced Practice Providers, St. Jude Children Research Hospital, Memphis, TN, United States; ^3^ Department of Pathology, St. Jude Children Research Hospital, Memphis, TN, United States; ^4^ Department of Pediatrics, University of Tennessee Health Science Center, Memphis, TN, United States; ^5^ Department of Pharmacy and Pharmaceutical Sciences, St. Jude Children Research Hospital, Memphis, TN, United States

**Keywords:** coronavirus, immunocompromised, cancer, hematopoietic cell transplant, monoclonal antibodies

## Abstract

From 8 December 2021 to 26 January 2023, tixagevimab–cilgavimab (T-C) was authorized for pre-exposure prophylaxis of COVID-19. During this period, we used a multidisciplinary team to communicate, screen, approach, and administer T-C to eligible patients. Twenty-seven patients were eligible. Of these, 24 (88.9%) received at least one dose of T-C and three patients received two doses. Majority of patients were White, non-Hispanic, and women. Only two patients had COVID-19 prior to receiving T-C. Seventeen (70.8%) had received two or more doses of SARS-CoV-2 vaccine. No serious adverse events were noted. Seven patients developed SARS-CoV-2 infection within 180 days of receiving T-C (median 102 days; range 28–135), and only one patient developed severe COVID-19 requiring intensive mechanical ventilation in the intensive care unit.

## Introduction

Severe acute respiratory syndrome coronavirus 2 (SARS−CoV−2) originated as an acquired infection from a zoonotic source from Huanan wholesale seafood market, which sold poultry, snake, bats, and other farm animals in Wuhan, Central China (1). It initially presented as a respiratory illness in the area, followed by a rapid global spread, leading to the WHO to declare a pandemic on 11 March 2020 (1, 2). This virus is transmitted from human to human through direct, contact, and airborne transmission through respiratory secretions and aerosols causing coronavirus disease 2019 (COVID-19) (1). Over time, complications, such as post-acute (long) COVID-19 were recognized. This condition has hindered millions of individuals from normal life by causing chronic pain, brain fog, shortness of breath, chest pain, and intense fatigue (3, 4).

SARS-CoV-2 circulation continues, and immunocompromised individuals remain at risk of severe disease, complications, and poor outcomes associated to COVID-19 (5–9). In addition, patients with cancer or those undergoing transplant may experience delay in needed therapies, even in the setting of mild to moderate infection (10–15). Vaccines are the most effective way in preventing severe COVID-19. However, Umakanthan et al. showed that uptake and adherence to preventive public health measures, such as vaccines varied significantly based on variables such as age, gender, and education (16). Particularly in the United States (US), misinformation and disinformation led to a significant decline in vaccine acceptance (17–19). However, even if vaccinated, these patients have suboptimal responses to vaccination, and the use of monoclonals during periods of intense immunosuppression is an effective strategy in preventing severe COVID-19 (20–23).

Tixagevimab–Cilgavimab (T-C; Evushled™, AstraZeneca, Wilmington, DE) is a combination of two long-acting neutralizing mAbs that bind to distinct epitopes of the SARS-CoV-2 spike-protein receptor-binding domain (24). T-C was first authorized for COVID-19 pre-exposure prophylaxis (PrEP) on 8 December 2021, in patients aged ≥ 12 years who weigh ≥ 40 kg and either (1) have moderate to severe immunosuppression and may not respond adequately to COVID-19 vaccination or (2) cannot receive any available COVID-19 vaccine due to a history of severe adverse reaction to a COVID-19 vaccine or its components. It was not authorized for post-exposure prophylaxis (PEP) or treatment, and recipients could not be infected with or been recently exposed to SARS-CoV-2 (25). Subsequently, the Food and Drug Administration (FDA) announced on 26 January 2023 that the product was no longer authorized due to inactivity against > 90% of current SARS-CoV-2 variants (26). In the European Union, T-C remains authorized with similar indications as in the US for PrEP, as well as for treatment who do not require supplemental oxygen and who are at increased risk of progressing to severe COVID-19 (27).

T-C was safe, well-tolerated, and prevented symptomatic COVID-19 due to susceptible variants (24, 28). In a placebo-controlled trial among adults, the incidence adverse events (AEs) observed did not differ between those who received T-C *versus* placebo (24, 25). Furthermore, T-C has been reported to reduce relative risk of symptomatic COVID-19 among adults who were at risk of poor vaccination response, higher SARS-CoV-2 exposure risk, or both (24). Post-EUA (Emergency Use Authorization) studies in adults with hematological malignancies or receiving solid organ transplantation confirm these findings (29–35). A recent meta-analysis reported overall clinical effectiveness of T-C among immunocompromised adults against COVID-19 hospitalization, intensive care admission, and COVID-19–specific mortality of 66.19, 82.13, and 92.39%, respectively (35). Despite these promising results among adults, no published data are available regarding the efficacy and safety of T-C among pediatric patients.

While T-C is currently not authorized in the US, it may become available in the future based on variant’s circulation. In addition, T-C remains authorized in other parts of the world, and new long-acting monoclonals that are only tested in adults may be authorized for those over 12 years of age or > 40 kg. Therefore, data on the safety and tolerability of T-C in children remain relevant. Furthermore, the described model of a multidisciplinary team promptly responding to changes in regulation and available therapeutics could be replicated by others to facilitate rapid administration of potential lifesaving interventions in high-risk patients.

## Methods

### Design and implementation

This is a single-center observational study performed at St. Jude Children’s Research Hospital (St. Jude). St. Jude is a research hospital specializing in the care of children with cancer, sickle cell disease, stem cell transplantation, HIV infection, and other non-malignant hematologic conditions, located in Memphis, TN, US.

In response to the EUA issued for T-C in December 2021, a multidisciplinary team composed of clinical pharmacists and pediatric infectious diseases, representing the institutional antimicrobial stewardship program; the infection, prevention, and control program; and leadership, was created to create an allocation program for T-C. Given the limited supply of T-C early on, a priority list was developed based on expert opinion and known risk factors for severe disease to ensure those patients who would benefit the most from T-C received the limited supply. Patients aged ≥ 12 years who weigh ≥ 40 kg who have moderate or severe immunocompromise resulting from qualifying conditions or receipt of immunosuppressive treatments, as well as individuals with allergic reactions to COVID-19 vaccines. Eligibility criteria included patients with any of the following: (1) de novo acute myeloid leukemia (AML) patients on therapy, (2) de novo acute lymphoblastic leukemia (ALL) patients receiving induction therapy, (3) relapsed/refractory AML or ALL patients on therapy, (4) recipients of allogeneic hematopoietic cell transplant (HCT) or chimeric antigen receptor (CAR) T-cell therapy within first 100 days, (5) bone marrow failure patients with B-cell aplasia or on immunosuppressive therapy; and (6) documented severe Polyethylene glycol (PEG) allergy. Second, we communicated to all clinicians in the institution using an SBAR (situation, background, assessment, and recommendation) email to raise awareness of eligibility criteria, safety, and efficacy of T-C (**Supplementary Table S1**). Following this, patients underwent daily eligibility screening by clinical pharmacists and advanced practice providers from the Infectious Diseases Department, and if patients met eligibility criteria, these were communicated with patients’ primary physicians. If in agreement, the medication was offered to patients, and caregivers were educated regarding treatment-C per EUA.

### Variables analyzed

T-C–related side effects were recorded, and SARS-CoV-2 infection post-administrations were captured retrospectively by pediatric infectious diseases physicians, using the electronic medical record. In addition, demographics (gender, age, race, and ethnicity), clinical information [including baseline diagnosis, reason for receiving T-C, previous COVID-19, vaccination history (type and number of doses)], time from previous COVID-19 to receiving T-C, time to COVID-19 from receiving T-C, and severity of COVID-19 (symptoms, hospitalization, progression to pneumonia, need for intensive care unit admission, mechanical ventilation, and death were obtained. Data are presented as frequency (%) for categorical variables and median (range) for continuous variables. The study was reviewed and approved by the St. Jude IRB with a waiver of informed consent. To ensure that data were unidentifiable age, time of SARS-COV-2 infection from T-C and time from transplant at the time of SARS-CoV-2 infection (if applicable) are presented as ranges.

## Results

### Participants

A total of 27 patients were deemed high risk for severe COVID-19 and eligible to receive T-C with 24 (88.9%) receiving at least one dose and three patients receiving a second dose over 180 days after the first dose. Two patients (8.3%) had COVID-19 prior to receiving the first dose of T-C (one 6 days prior and the other 121 days prior). All patients received T-C due to moderate to severe immunosuppression, with the majority (54.16%) being within 100 days following allogeneic HCT (**Table 1**). Among the three T-C eligible patients who did not receive T-C, one was deferred due to end-of-life care and two opted instead to only receive SARS-CoV-2 vaccination.

**Table 1:** Demographics of immunocompromised pediatric patients who received T-C.

Variables		n=24
**Age (median [range])**		16.50 [12 – 21]
**Gender (%)**	Female	14 (58.3)
	Male	10 (41.7)
**Race (%)**	Black or African American	7 (29.2)
	Other	2 (8.3)
	White	15 (62.5)
**Ethnicity (%)**	Hispanic	7 (29.2)
	Not Hispanic	17 (70.8)
**Diagnosis (%)**	Mixed phenotype leukemia	1 (4.2)
	ALL	6 (25.0)
	AML	7 (29.2)
	Hematologic Disease	10 (41.7)
**Reason to receive T-C**	< 100 days from allogenic HCT Graft versus host disease Post CAR-T therapy Chemotherapy Other*	13 (54.2) 4 (16.7) 3 (12.5) 2 (8.3) 2 (8.3)
**SARS-CoV-2 Vaccination Received**		
**Pfizer-BioNTech (%)**	No	4 (16.7)
	Yes	20 (83.3)
**Moderna (%)**	No	15 (62.5)
	Yes	9 (37.5)
**Janssen (%)**	No	20 (83.3)
	Yes	4 (16.7)
**Received > 2 doses of vaccine (%)**	No	7 (29.2)
	Yes	17 (70.8)
**Number of vaccine doses/patient before T-C (median [range])**		2 [0 – 3]
**SARS-CoV-2 infection before T-C dose infection, no. (%)**	No	22 (91.7)
	Yes	2 (8.3)
**Time between SARS-CoV-2 infection and first dose T-C (median [range])**		63.50 [6–121]
**Platelet count prior to any T-C dose (median [range] 10^3^/ mm^3^)**		170 [25-561]
**SARS-CoV-2 infection <=180 days from any T-C dose**	No	17 (70.8)
	Yes	7 (29.2)
**Time between any dose T-C and SARS-CoV-2 infection (median [range])**		102 [28–135]

*Other: Lupus on immunosuppressive therapy (n=1); Kidney transplant recipient on immunosuppressive therapy ^ Hematologic diseases: Aplastic Anemia (n=4), Sickle Cell Disease (n=4), Acquired Factor II deficiency (n=1), Myelodysplastic Syndrome (n=1).

### Safety

Thrombocytopenia with risk for hematoma was a concern prior to administration in two patients with platelets below 50 10^3^/mm; however, none developed a hematoma. All patients tolerated T-C without serious AEs or cardiac complications. The most common AE was immediate injection site pain. There were mild and resolved without intervention.

### Efficacy

Seven patients (29.2%) had SARS-CoV-2 infection within 180 days following T-C administration: six patients after the first dose and one after the second dose. The median time from T-C administration to SARS-CoV-2 infection was 102 days (range: 28–135), and for all seven, this was their first episode of COVID-19 recorded (**Table 2**). All seven patients had SARS-CoV-2 in 2022, during which time Omicron accounted for > 99% of all variants characterized in the US. Case summaries of these seven patients follow:

Patient 1. A 14- to 17-year-old man with relapsed ALL (between days +200 and +250 from haploidentical transplant) who tested positive for SARS-CoV-2 on asymptomatic screening test between 100 and 130 days after receiving T-C. He had received three doses of mRNA SARS-CoV-2 vaccine, with the last dose given within a month prior to infection. He reported upper respiratory symptoms 10 days prior to diagnosis but did not seek care. The patient did not develop Lower respiratory tract infection (LRTI) and fully recovered.
Patient 2. A 14- to 17-year-old woman with AML (between days +100 and +150 from match unrelated donor transplant) who tested positive for SARS-CoV-2 in setting of mild, non-productive cough, between 100 and 130 days after receiving T-C. The patient had received three doses of mRNA vaccine; the last was given > 6 months prior to infection. She did not develop LRTI and fully recovered.
Patient 3. A 14- to 17-year-old woman with AML (between days +100 and +150 from haploidentical transplant) who tested positive for SARS-CoV-2 in setting of mild Upper respiratory tract infection (URTI) symptoms, between 70 and 100 days after receiving T-C. Patient had not received any vaccine doses prior to this episode. She did not develop LRTI and fully recovered.
Patient 4. A 14- to 17-year-old man with mixed phenotype leukemia on chemotherapy who tested positive for SARS-CoV-2 in setting of mild URTI symptoms, between 120 and 150 days after receiving T-C. The patient had received four doses of mRNA vaccine; the last was given 5 months prior to this episode. He did not develop LRTI and fully recovered.
Patient 5. A 13- to 16-year-old woman with history of AML (between days +10 and +150 from haploidentical transplant) who tested positive for SARS-CoV-2 through asymptomatic screening, between 60 and 90 days after receiving T-C. Patient had received three doses of mRNA vaccine; the last was given 48h prior to this episode. He did not develop LRTI and fully recovered.
Patient 6. A 12- to 15-year-old man with B-cell ALL and persistent B-cell aplasia post-autologous CAR–T-cell therapy. He received two doses of T-C. He did not develop SARS-CoV-2 infection within 180 days of the first dose but tested positive between 15 and 30 days after the second dose through an asymptomatic screening test. He had received two doses of mRNA vaccine; the last was given over a year prior to infection. The patient did not develop LRTI and fully recovered.
Patient 7. A 17- to 20-year-old man with history of AML (over 5 years after second haploidentical transplant) with bronchiolitis obliterans (BOS) who developed two episodes of SARS-CoV-2 infection after the first dose of T-C. Due to his lung disease, he previously required hospitalization on several occasions for respiratory failure in the setting of viral infection. He received two doses of Janssen COVID-19 vaccine; the last was given > 7 months prior to the first SARS-CoV-2 infection. His first SAR-CoV-2 infection was between 80 and 110 days after the first T-C dose. He received 3 days of remdesivir, did not require hospitalization, and fully recovered. He tested positive again 10 days after initial positive testing, and eventually had two negative RT-PCR tests 24h apart, a month after initial positive testing. Shortly after, the first episode, he again tested positive for SARS-CoV-2 (between 120–150 days after the first dose of T-C). With this episode, he developed respiratory failure requiring ICU admission and mechanical ventilation. SARS-CoV-2 was detected by nasopharyngeal swab and bronchoalveolar lavage. This episode occurred at a time when BA.5 was the predominant SARS-CoV-2 variant in the USA. In addition, Bronchoalveolar lavage (BAL) was positive for rhino/enterovirus and HSV-1. He received 10 days of remdesivir, methylprednisolone 2 mg/kg, and baricitinib for 14 days, as well as IV acyclovir. He improved and was discharged from the hospital after 1 month. He received a second dose of T-C 6 months after first dose (1 month after severe COVID-19 infection) and a dose of mRNA vaccine. He did not have another SARS-CoV-2 episode during the 6 months after his second dose of T-C.

**Table 2:** Characteristics of high-risk patients with SARS-CoV-2 infection within 180 days of T-C.

Patients	Diagnosis	Age (years)*	Gender	# T-C doses	Predominant variant at the time of infection (27)	Time from T-C to SARS-CoV-2 infection (days)*	Vaccinated prior to SARS-CoV-2 infection	Severe COVID-19
1	relapsed ALL	14-17	M	1	BA.5	100-130	Yes	No
2	AML	14-17	F	1	BA.5	100-130	No	No
3	AML	14-17	F	1	BA.5	70-100	Yes	No
4	MPL	14-17	M	1	BA.5	120-150	Yes	No
5	AML	13-16	M	1	BQ.1	60-90	Yes	No
6	ALL	12-15	M	2	BA.5	15-30	Yes	No
7^	AML	17-20	M	2	BA.5	80-110	Yes	Yes

^ Patient had a second episode of SARS-CoV-2 between 120-150 days after first dose of T-C. ALL, acute lymphoblastic leukemia; AML, acute myeloid leukemia; MPL, mixed phenotype leukemia. * To ensure that data was unidentifiable age and time of SARS-CoV-2 infection from T-C dose are presented as ranges.

## Discussion

This is the first report on the safety and tolerability of T-C in children. The management of COVID-19 evolved rapidly during the pandemic. While vaccination effectively decreases severe disease, immunocompromised patients remain at risk even if they have received all recommended doses (20–23, 36, 37). Available antivirals can decrease progression to pneumonia in patients at high risk; however, they are most efficacious when early after infection and their utility depends on prompt diagnosis. In addition, some centers do not have capacity for outpatient remdesivir administration as well as the combination of nirmatrelvir and ritonavir is sometimes contraindicated, usually because of drug–drug interactions (38–41). These limitations highlight the importance of having a long-acting monoclonal antibody for PrEP in our arsenal against COVID-19.

Consistent with previous literature published among adults, no serious AEs were observed among our pediatric patients after receiving T-C (29, 30, 42). Although our study was not designed to test statistical significance, most cases included in our report who received T-C did not have COVID-19 within 6 months of administration. Furthermore, while seven patients had COVID-19 post–T-C, only one patient (14%) developed severe COVID-19, and this occurred during a period when BA.5 was the most prevalent variant of concern circulating in the US and several reports showed decrease activity of this monoclonal antibody (34, 43, 44), eventually leading to the US FDA to revoke the EUA (26). In addition, the episode of severe disease occurred 139 after the first dose, close to the 180 days recommendation for re-dosing. Whether this was related to weaning antibody levels, suggesting that some patients may benefit from more frequent dosing, due to other infections or host characteristics or to infection by a strain resistant to T-C remains unknown.

### Strengths and limitations

This study had several limitations. First, it was a single-center experience with relatively few patients describing the implementation of T-C EUA clinically. Second, the follow-up period was inconsistent, and the information was obtained retrospectively through medical chart review. Despite these limitations, this study is one of the first to provide valuable data about pediatric patients who received T-C, with a detailed description of their COVID-19 after T-C administration.

### Conclusion and clinical significance

Data on the safety, tolerability, and efficacy of monoclonal antibodies against SARS-CoV-2 in children remains scarce. Changes in dominant variants over time have consistently led to early closure of pediatric trials. Furthermore, most monoclonal antibodies active against SARS-CoV-2 were limited to individuals greater than 12 years of age. Emphasis should be placed on pediatric trials with younger aged cohorts to access the feasibility and efficacy evidence-based interventions in this group. In the meantime, retrospective description of institutional experiences, such as this one, can increase our understanding of the safety and utility of monoclonal antibodies against SARS-CoV-2 in this population and provide valuable data for designing future prospective clinical trials.

## Data availability statement

The raw data supporting the conclusions of this article will be made available by the authors, without undue reservation.

## Ethics statement

The studies involving human participants were reviewed and approved by St Jude Children’s Research Hospital IRB. Written informed consent from the participants’ legal guardian/next of kin was not required to participate in this study in accordance with the national legislation and the institutional requirements.

## Author contributions

DH and SC conceptualized and designed the study. DH, RD and SC drafted the initial manuscript and reviewed and revised the manuscript. JW, WG, TM, and SC coordinated and supervised data collection, and critically reviewed the manuscript for important intellectual content. ES, CM and AM collected the data. RD and JF designed the data collection instruments, carried out the initial analyses, and reviewed and revised the manuscript. All authors approved the final manuscript as submitted and agreed to be accountable for all aspects of the work.
