# Supplementary material for: SARS-CoV-2 infection in high-risk children following tixagevimab–cilgavimab (Evusheld) pre-exposure prophylaxis: a single-center observational study
Source: Front Oncol. 2023 Aug 3;13:1229655. doi: 10.3389/fonc.2023.1229655 (PMC10436088; doi:10.3389/fonc.2023.1229655)
Supplement: Supplementary file 1 [file Table_1.docx]

**Supplementary Table 1:** **SBAR effective 2/23/2022: Evusheld™ (tixagevimab and cilgavimab) for pre-exposure prophylaxis of COVID-19 in certain high-risk patients over 12 years of age who weigh at least 40 kg**

| Situation | SARS-CoV-2 vaccines remain the most effective way of preventing COVID-19. However, the effectiveness of these vaccines remains diminished in those who are immunosuppressed. |
| --- | --- |
| Background | Immunocompromise may prevent an optimal immune response to vaccination against SARS-CoV-2 leading to persistent susceptibility to severe illness. In addition, some patients are unable to be vaccinated.  The newly available monoclonal antibodies tixagevimab and cilgavimab (co-packaged as Evusheld™) bind to non-overlapping regions of the spike protein receptor binding domain and have a long half-life of about 3 months. These antibodies retain activity against the omicron variant.  Evusheld™, when used as pre-exposure prophylaxis led to a 77% relative risk reduction in symptomatic COVID-19 with protection from the virus continuing for at least six months. It has been granted FDA EUA for patients ≥ 12 years of age who have contraindications to COVID-19 vaccination OR who may not mount an immune response to vaccinations.  Pre-exposure prophylaxis with Evusheld™ **is not a substitute for vaccination in individuals for whom COVID-19 vaccination is recommended.** Individuals for whom COVID-19 vaccination is recommended, including individuals with moderate to severe immune compromise, should receive COVID-19 vaccination.  **Evusheld™ is not authorized for post-exposure prophylaxis or treatment of COVID-19.** |
| Assessment | St Jude patients over 12 years of age (and ≥ 40 kg weight) who are severely immunosuppressed or those with severe anaphylactic reaction to PEG are eligible and would benefit from this product. |
| Recommendation | The EUA specifies use of Evusheld™ as pre-exposure prophylaxis in those aged ≥12 years who weigh ≥40 kg who have moderate or severe immunocompromise resulting from qualifying conditions or receipt of immunosuppressive treatments. In addition, use is allowed in those with allergic reactions to COVID-19 vaccines.  The following groups of patients should be considered for Evusheld™ administration:   - De novo AML patients on therapy - De novo ALL patients receiving induction therapy. - Relapsed/refractory AML or ALL patients on therapy - Recipients of allogeneic HCT or CAR T-cell therapy within first 100 days - Bone marrow failure patients with B cell aplasia or on immunosuppressive therapy - Documented severe PEG allergy.   Other cases are considered on a case-by-case basis. Patients who are eligible for and receive Evusheld™ should also get COVID vaccine if indicated per St Jude guidelines.  Tixagevimab and cilgavimab are administered as **two intramuscular 1.5 mL** injections. Additional doses may be given at 6-month intervals (although supporting data are not yet available).  Members of the Antimicrobial Utilization and Improvement Committee have begun a targeted search for potentially eligible St. Jude patients and will contact attending physicians of identified patients.  If you think your patient would be a candidate for Evusheld™ or have questions about this SBAR, please contact pharmaceutical services.  Additional information can be found here:   1. Evusheld FDA Emergency Use Authorization 2. Evusheld Fact Sheet for Healthcare providers 3. Evusheld Fact Sheet for patients, parents and caregivers |
